# Supplementary figures and images for: Comparison of a novel potentiator of CFTR channel activity to ivacaftor in ameliorating mucostasis caused by cigarette smoke in primary human bronchial airway epithelial cells
Source: Respir Res. 2024 Jul 10;25:269. doi: 10.1186/s12931-024-02889-w (PMC11234710; doi:10.1186/s12931-024-02889-w)

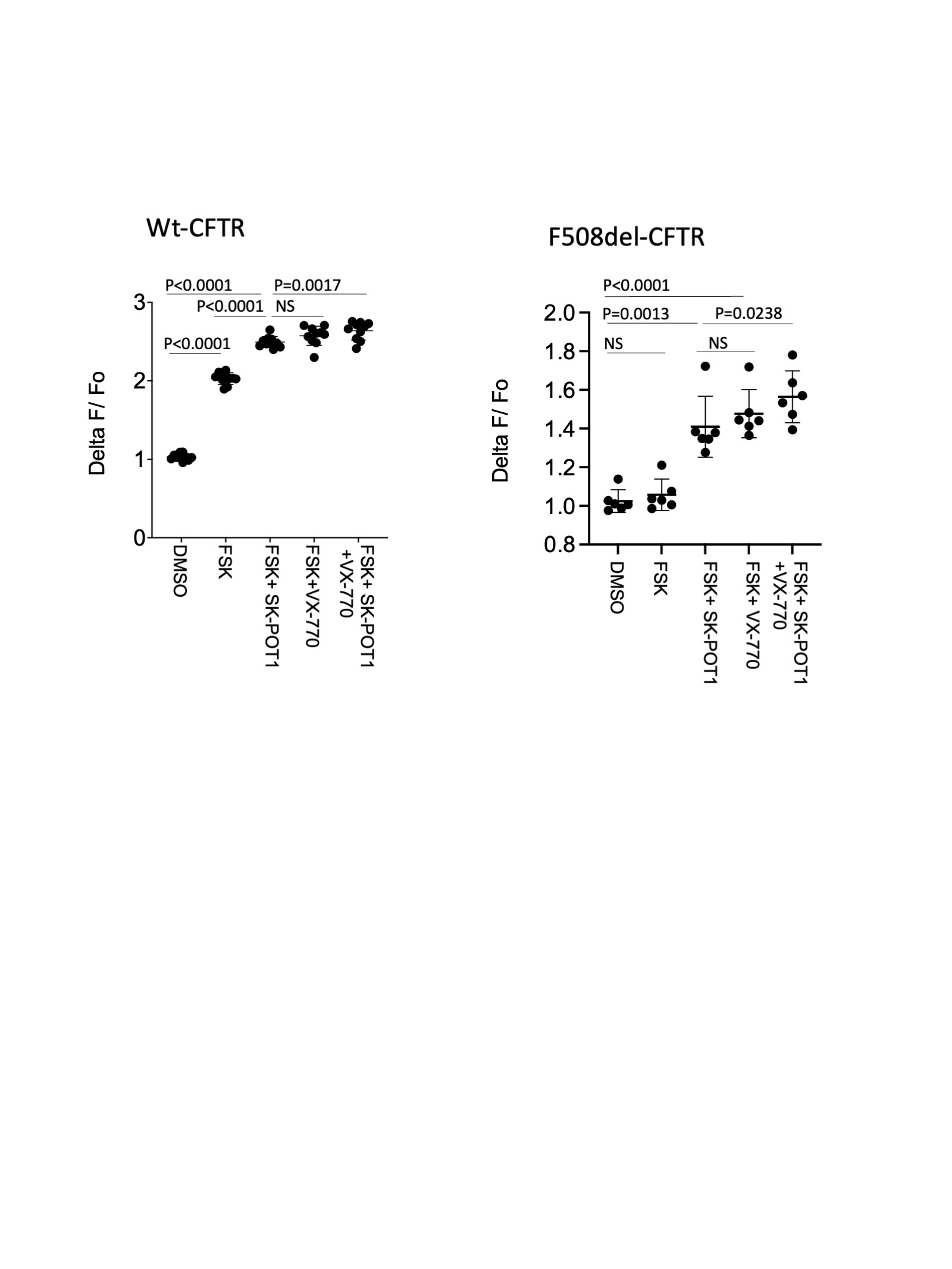

Supplement: Supplementary file 1 — Supplementary Material 1. Lack of additive effect of VX-770 (0.1µM) and SK-POT (0.1µM) in potentiating forskolin activated Wt-CFTR channel function or F508del-CFTR (corrected using 3 µM VX-661). Wt or F508del-CFTR were stably expressed in HEK cells. Wt-CFTR was activated using 1 µM forskolin and VX-661 corrected F508del, activated with 10 µM forskolin. For Wt-CFTR and F508del, each symbol reflects a peak response from a sample (well) generated from a total of 3 biological replicates (or cell platings). Mean and SD shown. Mean and SD shown. For Wt and F508del-CFTR, datasets were compared using one-way ANOVA with Tukey’s post-hoc multiple comparison test. [file 12931_2024_2889_MOESM1_ESM.jpeg]

## Slide 1
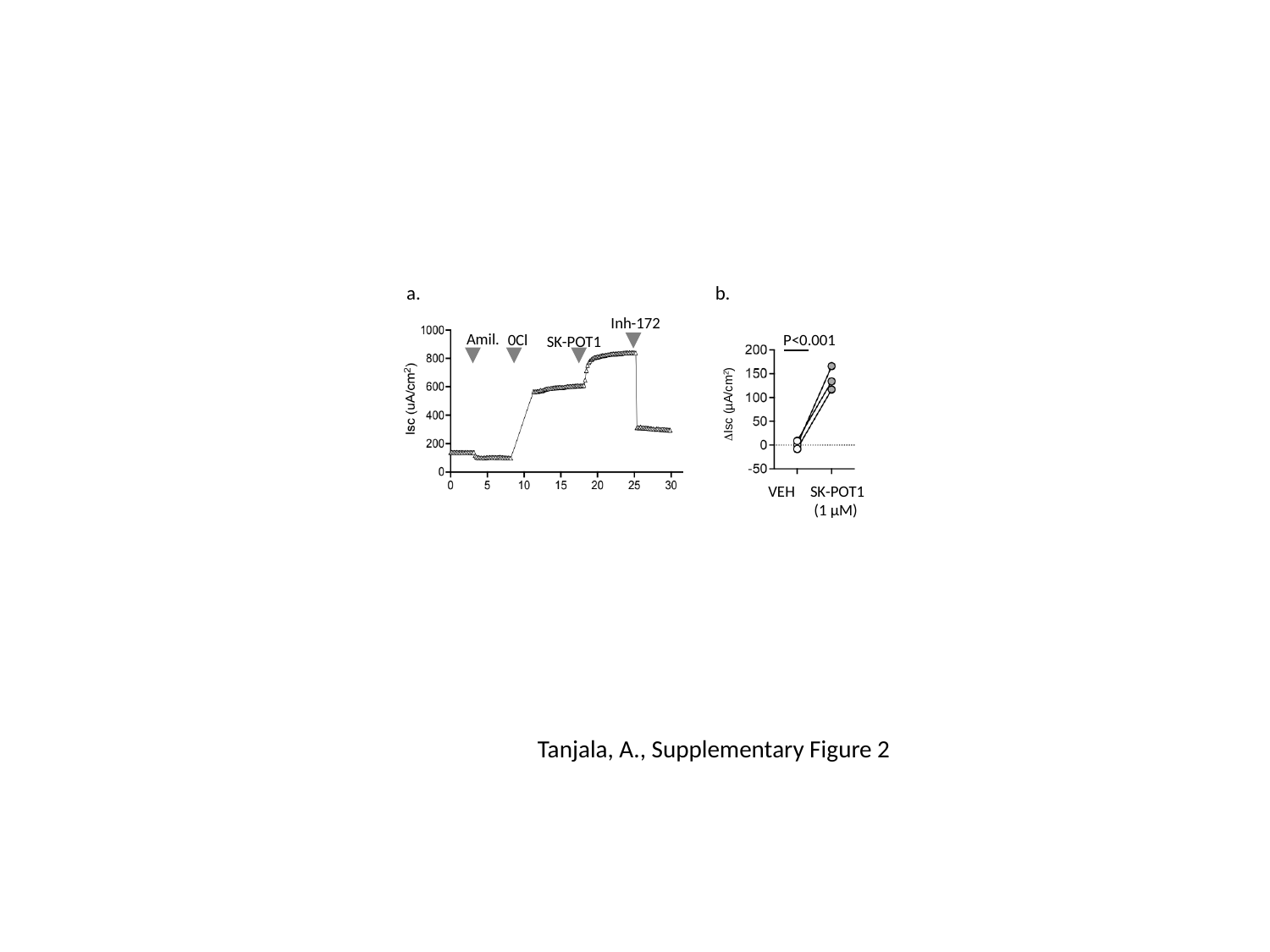

a.
b.
Inh-172
Amil.
P<0.001
0Cl
SK-POT1
DIsc (µA/cm2)
VEH
SK-POT1
 (1 µM)
Tanjala, A., Supplementary Figure 2

Supplement: Supplementary file 2 — Supplementary Material 2. Representative Ussing chamber studies of SK-POT1 (1 µM) potentiation after forskolin activation (100 nM) in primary bronchial epithelial cultures prepared from adult ferret trachea. ii. Scattergram shows forskolin-dependent changes in CFTR mediated short circuit with the addition of SK-POT1 or VEH (n=3 ferret bronchial cultures). Student’s “t” test was conducted. [file 12931_2024_2889_MOESM2_ESM.pptx]
